# A fast and fully automated system for glaucoma detection using color fundus photographs

**DOI:** 10.1038/s41598-023-44473-0

**Published:** 2023-10-27

**Authors:** Sajib Saha, Janardhan Vignarajan, Shaun Frost

**Affiliations:** Australian e-Health Research Centre, Commonwealth Scientific and Industrial Research Organisation (CSIRO), Perth, Australia

**Keywords:** Image processing, Machine learning, Translational research, Computer science

## Abstract

This paper presents a low computationally intensive and memory efficient convolutional neural network (CNN)-based fully automated system for detection of glaucoma, a leading cause of irreversible blindness worldwide. Using color fundus photographs, the system detects glaucoma in two steps. In the first step, the optic disc region is determined relying upon You Only Look Once (YOLO) CNN architecture. In the second step classification of ‘glaucomatous’ and ‘non-glaucomatous’ is performed using MobileNet architecture. A simplified version of the original YOLO net, specific to the context, is also proposed. Extensive experiments are conducted using seven state-of-the-art CNNs with varying computational intensity, namely, MobileNetV2, MobileNetV3, Custom ResNet, InceptionV3, ResNet50, 18-Layer CNN and InceptionResNetV2. A total of 6671 fundus images collected from seven publicly available glaucoma datasets are used for the experiment. The system achieves an accuracy and F1 score of 97.4% and 97.3%, with sensitivity, specificity, and AUC of respectively 97.5%, 97.2%, 99.3%. These findings are comparable with the best reported methods in the literature. With comparable or better performance, the proposed system produces significantly faster decisions and drastically minimizes the resource requirement. For example, the proposed system requires 12 times less memory in comparison to ResNes50, and produces 2 times faster decisions. With significantly less memory efficient and faster processing, the proposed system has the capability to be directly embedded into resource limited devices such as portable fundus cameras.

## Introduction

Glaucoma is a neurodegenerative disease of the eye, which affects the optic nerve head (ONH)^1^. Glaucoma in its early stage does not produce noticeable symptoms or changes to the visual field^2^. However, as the disease progresses, a gradual narrowing of visual field is typically observed, starting from the periphery and extending towards the center. If not treated in a timely fashion, glaucoma can lead to complete blindness^3–5^.

There are 80 million people worldwide with glaucoma, and this number is projected to reach more than 111 million by 2040^6^. Glaucoma is also responsible for blindness in approximately 4.5 million people worldwide, making it the second main cause of complete vision loss worldwide^7^. Whilst aging, and ethnicity are some of the important risk factors of glaucoma, unfortunately, the disease is present irrespective any age group or population type, and both developed and developing countries^8^. Because of its asymptomatic nature in early stages, it is reported that approximately 50% of patients are unaware of the disease^9^. However, to save vision in glaucoma it is important to intervene early; research shows that in 50% cases, early intervention can delay the onset of blindness in glaucoma by 20 years^10^. Therefore, early screening and diagnosis support is crucial. Automated assessment of glaucoma is a cost-effective way for the early diagnosis and management of the disease.

In addition to visual field testing, there are two other tests that are usually performed to detect glaucoma: (1) intraocular pressure (IOP) measurement, (2) optic nerve head (ONH) assessment. IOP is the eye's fluid pressure and is typically measured using a tonometer. While high IOP is generally indicative of glaucoma, it is not always present and is considered a risk factor (Access Economics, 2008). The ONH is the region in the retina where blood vessels and nerve fibers converge and through which visual information transits to the brain. The evaluation of the ONH could be done in a number of ways including standard imaging techniques such as color fundus photography (CFP) and optical coherence tomography. While optical coherence tomography provides a cross-sectional view and depth information, CFP is more often used in the context because of the lower cost and ease of interpretability. In CFP, the ONH appears as a bright circular region called the optic disc (OD), and inside the OD, a brighter region called the optic cup (OC) is apparent.

**Figure 1:**
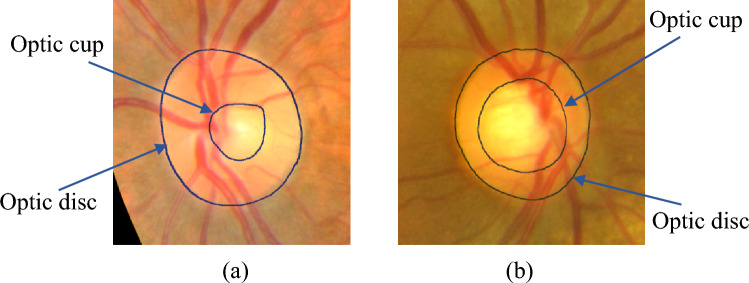
Example representation of (**a**) healthy, and (**b**) glaucomatous optic nerve head. Inner smaller circle is the optic cup and larger circle is the optic disc.

**Figure 2:**
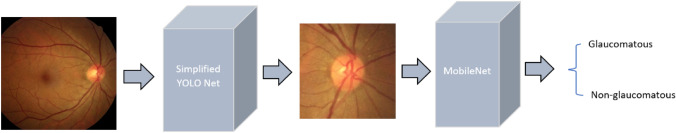
Proposed system architecture.

CFP highlights important parameters of the eye relevant to the disease, such as, the optic cup to disc ratio (CDR), peri-papillary atrophy (PPA) and neuroretinal rim loss. CDR, which measures the vertical diameter of the optic cup to that of the disc, is among one of the most commonly adopted screening tests for glaucoma^11^. Figure 1 shows example healthy (Fig. 2a) and glaucomatous ONH (Fig. 1b).

Manual assessment of optic nerve head is a time-consuming, tedious and subjective task, which necessitates the development of automated methods. In recent years there has been an increasing interest to develop deep learning, also known as convolutional neural network (CNN),-based methods, for automated assessment of glaucoma^12^. The main benefit of CNN-based methods is their ability to engage the machine to learn the inherent imaging features relevant to the disease by itself^13^. CNN-based methods have been found to be more than 90% accurate in detection of glaucoma, comparable to human experts^14,15^. While these methods are found to be highly accurate, the majority of them requires manual cropping of optic disc region, and importantly almost all of them are highly computationally intensive; which makes it harder to integrate them into portable fundus cameras.

In contrast to other works in the context, in this study we aim to develop a fully automated and low computationally intensive CNN-based system that could run effectively on resource limited devices such as the imaging device (i.e., camera) itself, and/or smartphones.

Specific contributions of this study include:

1. Development of a fully automated glaucoma detection system for resource limited devices.
2. A context dependent simplified version of YOLO Nano architecture, resulting improved performance.
3. Discovery of the fact that low-resource intensive CNNs are at least as good resource intensive CNNs in glaucoma.

## Related work

Automated glaucoma detection methods can be largely divided into two groups: (1) traditional rule-based machine learning methods, and (2) deep learning-based methods.

### Traditional rule-based machine learning methods

Traditional rule-based methods rely on predefined (or human crafted) features extracted from the image and fed into a machine learning classifier to generate a decision. The majority of traditional rule-based machine learning methods in glaucoma assessment focus on first segmenting the optic disc region^15–17^, and then training a classifier based on key statistical and/or physical features on this region.

In this section we review some of the recently proposed and well performing traditional rule-based machine learning methods. Septiarini et al.^18^ put forward a multi-step technique for glaucoma detection. The images were first preprocessed to remove noise. Then histogram equalization was applied to improve the quality of the images. Finally, K-NN classifier was applied to differentiate between the glaucomatous and healthy images. They achieved an accuracy of 95.24% on a dataset of 84 fundus images. Maheshwari et al.^19^ proposed an iterative approach for glaucoma detection. The images were first iteratively decomposed using variational mode decomposition (VMD) techniques. ReliefF algorithm was employed for features extraction, and LS-SVM classifier was used for classification of glaucomatous or healthy. They achieved an accuracy of 95.19%. Agrawal et al.^20^ proposed a multi-stage framework for glaucoma detection that include decomposition of the fundus images using the quasi bivariate variational mode decomposition (QB-VMD), followed by ReliefF method based feature extraction and finally SVM based classification. They achieved a tenfold cross validation accuracy of 86.13% on the RIM-ONE dataset. Kirar et al.^21^ proposed a glaucoma detection model using DWT and EWT based feature extraction. Support Vector Machine classifier was used for the classification of glaucomatous and healthy images. They achieved an accuracy of 83.57%, with sensitivity and specificity respectively 86.40%, and of 80.80%. Gour et al.^22^ proposed a multi-step model to detect glaucoma using the fundus images. The images were first preprocessed through CLAHE histogram equalization technique. Feature selection was then performed relying upon PCA. Finally, classification was performed by SVM. On the HRF data set, they achieved accuracy of 79.20% and AUC of 86%. Mookiah et al.^23^ used high-level statistical measures based on Fourier methods as primary features to be extracted for glaucoma assessment. An SVM was then trained on those features to classify glaucomatous against non-glaucomatous subjects. They achieved an accuracy of 95%, with sensitivity and specificity respectively 93.3% and 96.7%. Nayak et al. Sundaram et al.^24^ developed a system for automated eye disease prediction inclusive of glaucoma, using bag of visual words and support vector machine (SVM). Local features were extracted using Speeded Up Robust Feature (SURF), which were then clustered to obtain Bag of Features/Visual Words, and finally classification was performed using SVM. A maximum classification accuracy of 92% was obtained.

### Deep learning-based methods

Deep learning methods learn to extract complex features from the data by itself, without the need of predefined human crafted features. Convolutional neural network (CNN), a deep learning architecture, progressively learns more complex and abstract visual features from the images with increasing network depth. Because of superior performance over traditional rule-based machine learning methods, deep learning-based methods are becoming increasingly popular in recent years for biomedical image analysis including glaucoma assessment.

In this section we review the recent CNN-based methods for glaucoma detection using CFPs. Li et al.^25^ trained an InceptionV3 model from the scratch using a dataset collected privately from various clinical settings in China. A total of 39,745 fundus images were used for train and validation of the model. While a total of 48,116 images were initially selected for this study, 7371 images were excluded because of poor quality or location. ADAM optimiser with a learning rate of 2 × 10^−3^ was used. They used a batch size of 32. Christopher et al.^26^ independently trained three CNNs, namely—VGG16, Inception and ResNet50. A total of 14,822 fundus images were used. The images were collected from two prospective longitudinal studies, The African Descent and Glaucoma Evaluation Study and The Diagnostic Innovations in Glaucoma Study. Both transfer learning and learning from scratch approaches were experimented while training the models. They found ResNet50 as the best performing model for glaucoma detection, and transfer learning as the best approach to train the model.

Raghavendra et al.^27^ proposed a simple 18-layer CNN and applied for glaucoma detection. The model consists of 4 convolution blocks, a fully connected layer followed by soft-max classifier. Each convolution block contains 1 convolutional layer, 1 batch normalization layer, 1 ReLU and 1 max-pooling layer. Stochastic gradient descent (SGD) optimiser was used and the learning rate was trialed logarithmically from 10^−1^ to 10^−4^. A total of 1426 fundus images collected from India was used in the experiment.

Al-Bander et al.^28^ trained a DenseNet model using four publicly available data sets for detecting glaucoma. Pal et al.^29^ developed specific CNN-based framework for glaucoma detection namely G-EyeNet. Liu et al.^30^ developed a system relying upon ResNet. A list of preprocessing that are specific to glaucoma detection including image down-sampling, generation of optic disc centered images, were performed. SGD was used as the optimizer for the gradient propagation. A total of 269,601 fundus images collected from Chinese Glaucoma Study Alliance were used for training and validation of the system. Diaz-Pinto et al.^14^ experimented on 5 different CNN architectures, namely, VGG16, ResNet, VGG19, Inception-V3, and Xception to assess glaucoma. The data set scans were cropped to get the OD regions. They obtained the best performance using Xception model. Commonly used pretrained models were used in the context by a list of authors; to mention a few, Gómez-Valverde et al.^31^, Phan et al.^32^.

Elangovan et al.^33^ proposed an 18-layer CNN model to assess glaucoma. Publicly available data sets, namely, RIM-ONE2, DRISHTI-GS1, ACRIMA, ORIGA, LAG, were used for the experiments. Sreng et al.^34^ developed a multi-stage framework for glaucoma detection. Initially, a DeepLabV3+ architecture segmented the optic disk. Afterwards, pre-trained CNNs were applied for feature extraction, and SVM was employed for the classification of glaucomatous and the healthy images. Srinivasa et al.^35^ presented a 8-layer model for the detection of glaucoma. The model consists of 8 layers inclusive of 4 convolutional, and 4 connected layers.

Gheisari et al.^36^ developed a model that combines CNN with the recurrent neural network for the enhanced glaucoma detection. They found improved glaucoma detection performance when temporal features, extracted from video, are also used in the detection along with the spatial features. They experimented with both VGG16 and ResNet models. Maheswari et al.^37^ developed a technique for glaucoma diagnosis using deep learning and local descriptors-based augmentation. Chaudhary et al.^38^ proposed a method, namely two dimensional Fourier–Bessel series expansion based empirical wavelet transform (2D-FBSE-EWT) for boundaries detection, and applied that for the decomposition of fundus images into sub-images. Glaucoma detection from sub-images were performed relying upon two methods: (1) Using conventional machine learning, (2) deep learning (ensemble ResNet-50).

Carvalho et al.^39^ applied three-dimensional convolutional neural network (3DCNN) for diagnosis of glaucomatous and healthy fundus images. They developed a technique which converts two-dimensional fundus images to three-dimensional volumes to be used by the 3DCNN. Lin et al.^40^ developed an algorithm named GlaucomaNet to classify glaucomatous and healthy eyes. GlaucomaNet consists of two convolutional neural networks, one performing preliminary grading and the other performing detailed grading. Fan et al.^41^ comprehensively explored the generalizability and explainability of Vision Transformer deep learning technique in detecting glaucoma using fundus photographs. Data-efficient image Transformer (DeiT), and ResNet-50 models were compared. Shoukat et al.^42^ trained ResNet model for the diagnosis of glaucoma. Image pre-processing is performed to enhance image quality prior to the assessment. Velpula et al.^43^ developed a multi-stage glaucoma classification model using pre-trained CNNs and voting based classifier fusion. They utilized 5 pre-trained CNNs, namely ResNet-50, AlexNet, VGG16, DenseNet-201 and Inception-ResNet-v2.

Table 1 summarises the overall performance of these models.

**Table 1:** Sensitivity, specificity, accuracy, and area under the curve (AUC) of different CNN-based methods in glaucoma detection.

Methods	Performance evaluation			
	Sensitivity (in %)	Specificity (in %)	Accuracy (in %)	Area under the curve (AUC) (in %)
Li et al.^25^	92.9	92.0	92.9	–
Christopher et al.^26^	92.0	93.0	–	97.0
Liu et al.^30^	96.2	97.7	–	99.6
Raghavendra et al.^27^	98.0	98.3	98.1	–
Al-Bander et al.^28^	78.5	99.8	87.6	–
Diaz-Pinto et al.^14^	93.0	86.0	96.0	
Gómez-Valverde et al.^31^	87.0	89.0	–	–
Phan et al.^32^	–	–	–	90.0
Elangovan et al.^33^	96.0	97.0	96.6	–
Sreng et al.^34^	–	–	99.7	–
Gheisari et al.^36^^,a^	71	76	–	–
Srinivasa et al.^35^	99.0	99.0	99.0	–
Chaudhary et al.^38^	91.1	94.3	91.1	83.3
Carvalho et al.^39^	85.5	80.9	83.2	83.2
Lin et al.^40^	–	–	95.0	–
Fan et al.^41^	–	–	–	91.0
Shoukat et al.^42^	97.3^b^	91.7	96.1	95.1
Velpula et al.^43^	–	–	97.3^c^	–

^a^Best performance when only spatial features are used.

^b^Overall.

^c^Overall, for binary classification.

## Methodology

### Dataset

Fundus images from seven publicly available datasets have been used in this study: large-scale attention based glaucoma (LAG) dataset^44^, ACRIMA^14^, Drishti-GS^45^, HRF^46^, RIM-ONE r2^47^, sjchoi86 HRF^48^ and DRIONS-DB^49^. A detailed breakdown of the datasets used in this study is provided in Table 2.

**Table 2:** Breakdown of the images available in each dataset.

Dataset	Glaucomatous	Non-glaucomatous	Total
LAG	1711	3143	4854
ACRIMA	396	309	705
Drishti-GS	70	31	101
HRF	15	30	45
RIM-ONE	194	261	455
Sjchoi86 HRF	101	300	401
DRIONS-DB	60	50	110
Total	2547	4124	6671
Total (after horizonal flipping)	4124	4124	8248

Excluding Drishti-GS and HRF, all datasets had image dimensionality less than 1000 × 1000 pixels. Among these datasets, only RIM-ONE images were provided cropped in the ONH regions. Since the ONH is the primary region of interest for glaucoma assessment^14,15^, in line, with other studies in the literature we manually cropped images from other datasets as well.

To overcome the class imbalance, present in the ‘glaucomatous’ and ‘non-glaucomatous’ images, and to balance them, a random subset of 1577 glaucomatous images are selected, horizontally flipped and are saved as new images.

Data augmentation, including − 5 to 5 degrees range rotation, shearing in the range of 0.2, and scaling in the range of 0.2, was performed, for both ‘glaucomatous’ and ‘non-glaucomatous’ images.

### Proposed system

A two-step approach for glaucoma detection using color fundus photograph is proposed. Given a fundus photograph as input, in the first step ONH region is detection relying upon simplified You Only Look Once (YOLO) neural network architecture^50^. In the second step the ONH region is classified into ‘glaucomatous’ or ‘non-glaucomatous’, relying upon MobileNetV3Small CNN^51^. The overall workflow of the system is shown in Fig. 2.

#### Step 1: optic nerve head (ONH) region detection

The ONH is the primary region of interest for glaucoma assessment. ONH area is only a fraction of fundus image, thus processing only the ONH region ensures the optimal use of the CNN’s learning capacity and improves the classification performance.

ONH region detection is performed based on YOLO architecture, which is extremely fast. In different to other competing region detection architecture such as Faster R-CNN^52^, YOLO utilizes features from all over the image to predict each bounding box. Further to that for an image, YOLO predicts all bounding boxes for all classes simultaneously. These design strategies enable YOLO to perform superfast object localization and comparatively less background mistakes than its competitors (e.g., R-CNN family of algorithms)^50^.

YOLO has a family of algorithms and in this study, we specifically focused on architectures that are designed for resource limited devices such as mobile phones. More particularly we focused on YOLO Nano^53^, and its variants namely, YOLO-v5 Nano^54^, and YOLO-v7 Tiny^55^, that are highly compact, super-fast and memory efficient algorithms. YOLO Nano^53^ architecture relies upon principal 3 concepts: (1) Residual Projection-Expansion-Projection Macroarchitecture, (2) Fully-connected Attention Macroarchitecture, and (3) Macroarchitecture and Microarchitecture Heterogeneity.

Residual Projection-Expansion-Projection (PEP) microarchitecture comprises of: (i) a projection layer, that projects output channels into a lower dimensional output tensor, (ii) an expansion layer, that expands the number of channels, (iii) a depth-wise convolution layer that performs spatial convolutions, and (iv) a projection layer that projects output channels into a lower dimension output tensor. 1 × 1 convolutions are applied in the all the layers, except the depth-wise convolution layer, where 3 × 3 convolution is applied. Residual PEP macroarchitectures enables significant reductions in computational cost without compromising model expressiveness.

The Fully-connected Attention (FCA) microarchitecture comprises of two fully-connected layers to learn the dynamic, non-linear inter-dependencies between channels. FCA produces modulation weights to re-weight the channels. FCA facilitates dynamic feature recalibration and ensures better utilization of the available network capacity.

The Macroarchitecture and Microarchitecture Heterogeneity in YOLO Nano architecture is presented through a diverse mix of PEP modules, EP modules, FCA, as well as individual 3 × 3 and 1 × 1 convolution layers, and in terms of each module or layer having unique microarchitectures. Heterogeneity in the YOLO Nano architecture helps to achieve a very strong balance between architectural and computational complexity and model expressiveness.

##### Simplified YOLO Nano

The original YOLO Nano^53^ follows the multi-scale prediction strategy of YOLO v3, or in other words, relies upon multiple-scale feature maps (13 × 13, 26 × 26, and 52 × 52 for input size of 416 × 416 pixels) to detect small objects. Among these feature maps, 52 × 52 feature map with the smallest receptive field is targeted for tiny objects^56^. For color fundus image, the ONH is not tiny, hence, the scale 52 × 52 of the original YOLO Nano could be hypothetically removed to speed up the calculation, without compromising the performance; and that is what has been done in this work.

#### Step 2: classification of ‘glaucomatous’ and ‘non-glaucomatous’

Classification of the ONH region (i.e., ‘glaucomatous’ and ‘non-glaucomatous’), is performed relying upon independently trained MobileNetV3Small model. Extensive experiments were conducted to determine optimal CNN in the context. Seven state-of-art CNNs with varying computational cost and memory requirements, namely ResNet50, InceptionV3, InceptionResNetV2, MobileNetV2, MobileNetV3Small, 18-Layer CNN, Custom ResNet were experimented. Training of the CNNs were performed both using “transfer learning” and “learning from scratch” approaches^57^.

*(1) Transfer learning:* Available pre-trained ImageNet weights were used to initialize the internal weights of all the models. Random initializations were used for the added top layers of the models. Each of the models were trained in 2 consecutive steps. First a partial training was performed, where all the layers of the model, except the newly added layers were frozen and training was performed for 5 epochs using RMSProp optimiser and a learning rate of 10^−4^. Afterwards, a full training of the model was performed. Full training was performed for 200 epochs for each of the models in consideration.

Five of the seven CNNs, who had pretrained weights available, were experimented. For each of the models, a number of fine-tuning strategies were experimented while training. More specifically, we performed experiment by fine-tuning of the top 0–100% of the layers with 10% step size. Both the ADAM and RMSProp optimisers were experimented. Different learning rates were also tested.

*(2) Learning from scratch:* All of the 7 models were experimented. For each of the models, random weights were used to initialize the model parameter, and a full training was performed, meaning all layers of the model are trained from the data. Glorot uniform function was used for all the models for initialization. We experimented on different learning rates and optimizers, namely ADAM and RMSProp. Training was performed for a total of 200 epochs for each model.

### Performance measurement

To evaluate the performance of ONH region detection task, intersection over union (IoU) score between the ground truth rectangle and the rectangle(s) returned by the system (i.e., Simplified YOLO Nano) was used. The IoU score is mathematically defined as:

$$ IoU_{G, O} = \frac{{\left| {G \cap O} \right|}}{{\left| {G \cup O} \right|}}, $$

where, *G* is the binary image containing the original rectangle (with inside filled), and *O* is the binary image containing rectangle(s) (with inside filled) returned by the system.

Dice coefficients, defined mathematically as below, was also used to evaluate the performance of the models.

$$ {\text{Dice }}\;{\text{coefficient}} = \frac{{2*\left| {G \cap O} \right|}}{\left| G \right| + \left| O \right|}, $$

where *G* and *O* were defined as above.

For the classification task sensitivity, specificity, accuracy, area under the curve (AUC) and F1 score were used. These metrics are defined mathematically as below.

$$ {\text{Sensitivity}} = \frac{TP}{{TP + FN}}, $$

$$ {\text{Specificity}} = \frac{TN}{{TN + FP}}, $$

$$ {\text{Accuracy}} = \frac{TP + TN}{{TP + TN + FP + FN}}, $$

$$ {\text{F1 }}\;{\text{score}} = 2\frac{{{\text{Precision}} \times {\text{Sensitivity}}}}{{{\text{Precision}} + {\text{Sensitivity}}}}, $$

where, $${\text{Precision}} = \frac{TP}{{TP + FP}}$$.

Here, *TP*, *TN*, *FP*, *FN* represent respectively true-positive, true-negative, false-positive, and false-negative results, respectively.

## Results

### ONH region detection

**Figure 3:**
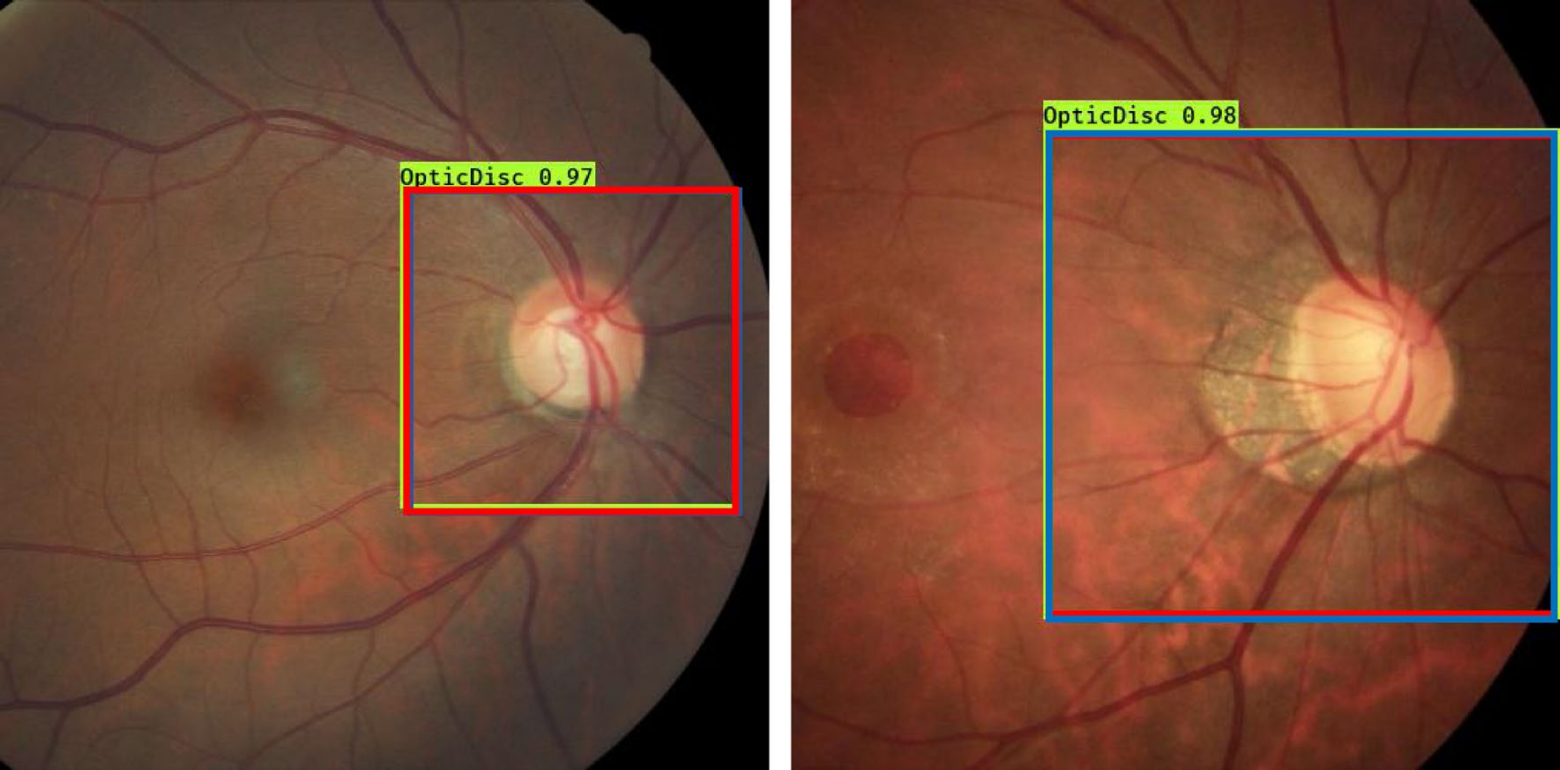
Example ONH bounding box determined by YOLO Nano and simplified YOLO Nano on 2 different example images. The ground truth bounding box is shown in “green”, bounding boxes determined by YOLO Nano and simplified YOLO Nano is respectively shown in “red” and “blue”.

Figure 3 shows example ONH detection by YOLO Nano and simplified YOLO Nano (proposed) model.

Table 3 quantitatively summarizes the performance of YOLO Nano, simplified YOLO Nano, YOLO-v5 Nano, and YOLO-v7 Tiny using dice coefficient and IoU score.

**Table 3:** Performance evaluation of YOLO Nano and simplified YOLO Nano.

Architecture	Model size (MB)	Dice co-efficient	IoU score
YOLO Nano	4	0.9045	0.8367
Simplified YOLO Nano	4	0.9103	0.8408
YOLO-v5 Nano^54^	4	0.9061	0.8391
YOLO-v7 Tiny^55^	12.6	0.9211	0.8610

From the results it is observable that YOLO-v7 Tiny marginally outperforms other models in terms of Dice co-efficient, IoU score; however, the model consumes 3 times more memory than others. Simplified YOLO Nano, YOLO Nano, and YOLO-v5 Nano all have very similar performance, with simplified YOLO Nano marginally outperforming YOLO Nano. Simplified YOLO Nano also requires about 3% less computation time than YOLO Nano.

### Classification of ‘glaucomatous’ and ‘non-glaucomatous’

Table 4 summarizes the performance of all the models for “transfer learning” approach, and Table 5 summarise the performance for “learning from scratch” approach.

**Table 4:** Performance evaluation of different models when transfer learning was used to train them. The values represented in this table are the average of the findings of tenfold cross validation.

CNN architecture	Sensitivity (%)	Specificity (%)	Accuracy (%)	AUC (%)	F1 score (%)
ResNet50	97.3	97.1	97.2	99.2	97.0
InceptionV3	94.7	95.1	95.0	98.4	94.5
InceptionResNetV2	96.3	97.5	97.2	99.3	96.9
MobileNetV2	95.1	97.2	95.6	99.0	96.1
MobileNetV3Small	97.5	97.2	97.4	99.3	97.3

**Table 5:** Performance evaluation of different models for “learning from scratch” approach. The values represented in this table are the average of the findings of tenfold cross validation.

CNN architecture	Sensitivity (%)	Specificity (%)	Accuracy (%)	AUC (%)	F1 score (%)
ResNet50	95.6	94.1	94.3	97.9	93.9
InceptionV3	95.4	94.6	94.6	98.1	94.8
InceptionResNetV2	96.1	94.7	94.6	98.2	95.0
MobileNetV2	94.6	94.2	94.6	98.1	94.4
MobileNetV3Small	95.2	95.3	95.2	98.4	95.1
18-Layer CNN	94.3	94.2	94.1	98.2	94.4
Custom ResNet	94.3	93.6	93.4	98.0	93.5

From Table 4, it is apparent that overall MobileNetV3Small outperforms all other models in consideration. In comparison to other models, MobileNetV3Small shows higher sensitivity, accuracy, AUC and F1 score, with comparable specificity. ResNet50 and InceptionResNetV2 are the next two models in terms of overall performance. ResNet50 shows higher sensitivity and F1 score in comparison to InceptionResNetV2, however, InceptionResNetV2 shows higher specificity and AUC. Both are found to produce identical accuracy. The difference in performance in any of these three models which include MobileNetV3Small, ResNet50 and InceptionResNetV2, are not to an extinct that would be relevant in practice.

From Table 5, it is apparent that overall MobileNetV3Small once again outperforms other models with higher specificity, accuracy, AUC and F1 score. InceptionResNetV2, InceptionV3 and ResNet50 shows marginally higher sensitivity than MobileNetV3Small. Following MobileNetV3Small, in terms of overall performance, InceptionResNetV2, InceptionV3 and ResNet50 are the next three models.

Unsurprisingly, with “transfer learning” higher performance of the models are observed in comparison to “learning from scratch”. Overall, a 0.8% increase in sensitivity, a 2.24% increase in specificity, a 1.82% increase in accuracy, a 0.9% increase in AUC and a 1.72% increase F1 score in comparison to “learning from scratch” approach is observable.

Table 6 summarizes the memory requirements, and time to produce decision by the different CNN models in consideration.

**Table 6:** Memory requirements and time to produce decision by the CNNs.

CNN architecture	No. of parameters (millions)	Time to produce decision (s)	Memory (MB)
ResNet50	25.6	82	98
InceptionV3	23.9	57	91
InceptionResNetV2	55.9	136	214
MobileNetV2	3.5	28	14
MobileNetV3Small	2.1	41	8
18-Layer CNN	4.4	103	17
Custom ResNet	5.2	14	20

From Table 6, it is apparent that MobileNetV3Small has the least memory requirement followed by MobileNetV2, 18-Layer CNN, and Custom ResNet. Custom ResNet requires the least amount of time to produce decision, followed by MobileNetV2 and MobileNetV3Small. ResNet50 requires 12 times more memory in comparison to MobileNetV3Small, and also takes twice the time of MobileNetV3Small to produce decision. The time and memory requirement for InceptionResNetV2 is respectively about 1.7 times and 2.2 times higher than ResNet50. InceptionV3 requires slightly less memory and time in comparison to ResNet50.

## Discussion

All the models were implemented in Keras (https://keras.io/) with a Tensorflow (https://www.tensorflow.org/) backend, excluding YOLO-v5 Nano and YOLO-v7 Tiny, which were implemented in PyTorch (https://pytorch.org/). Specific software package versions are: Python, version: 3.8.12, Keras, version: 2.4.3, Tensorflow, version: tensorflow-gpu-2.3.0, PyTorch, version 2.0.0.

The training, and validation was performed on a Dell Precision 5820 Tower Workstation. The workstation had Intel Xeon 3.60 GHz CPU, 64 GB RAM, and an NVIDIA GeForce RTX 2080Ti GPU (Dell Inc., Round Rock, TX, USA) installed.

With the exception of Custom ResNet and 18-Layer CNN, all other CNNs were readily available in Keras, and were used without alteration. Custom ResNet and 18-Layer CNN were implemented following the description and guideline of the authors. ADAM and RMSProp optimisers were independently experimented for all of the models in consideration. We also experimented with different learning rates in the range 10^−5^–10^−3^. The results reported in the tables are the results of the optimal setup.

We also experimented with detecting glaucoma without performing the ONH region detection. The aim of this experiment was to justify the requirement of region detection as a priory step for glaucoma detection. Without ONH region detection, the performance of all the models deteriorate. For high computationally intensive models (e.g., Inception-v3, ResNet50, InceptionResNet-v2) an overall accuracy decrease of 2% was observed. For low computationally intensive models the accuracy drop where about 3%. Table 7 shows the findings. Only the CNNs who had available pretrained weights were used in this experiment. Also, RIM-ONE dataset was excluded in this experiment, since only cropped images were available.

**Table 7:** Performance evaluation of different CNN models when glaucoma detection was performed on the whole images. The values represented in this table are the average of the findings of tenfold cross validation.

CNN architecture	Sensitivity (%)	Specificity (%)	Accuracy (%)	AUC (%)	F1 score (%)
ResNet50	95.1	96.2	96.1	98.1	96.2
InceptionV3	93.5	95.2	94.6	97.2	94.1
InceptionResNetV2	94.8	96.2	96.3	98.4	94.8
MobileNetV2	92.4	95.1	93.1	96.8	93.3
MobileNetV3Small	94.5	95.0	94.7	97.1	96.1

For ONH region detection, we experimented with YOLO Nano, YOLO-v5 Nano, and YOLO-v7 Tiny. We also proposed context aware simplification strategy and experimented with the original YOLO Nano. Similar context aware simplification strategy could also be adopted for YOLO-v5 Nano, and YOLO-v7 Tiny. However, we did not find any suitable implementation of YOLO-v5 Nano, and YOLO-v7 Tiny readily available that could be customized as per the need.

In this study a total of 6671 images were available for training and validation. In order to better investigate the performance and overall acceptability of the model, in line with the literature^58^, a tenfold cross validation was performed. The images had varying image dimensionality. However, experimentally we did not observe any difference in performance that would be relevant in practise attributed to this.

## Conclusions

Recent studies using CNN-based models for automated glaucoma detection have achieved performance comparable to human experts^59^. These studies limited their focus to performance, without considering the computational resource and/or time requirement to produce the decision. In this study we propose a fast and fully automated system for glaucoma detection, without compromising performance. The effectiveness of different CNNs architectures was experimentally evaluated using publicly available datasets. The proposed system relying upon simplified YOLO net (also proposed in this study) and MobileNetV3Small achieves an accuracy of 97.4% with sensitivity and specificity respectively 97.5% and 97.2%. The performance of the proposed system is at least as good as the highly computationally intensive state-of-the-art CNNs, experimented in this study, and also reported in the literature (Table 1). The best reported method in the literature produced an accuracy, sensitivity and specificity of respectively 99%, 99% and 99%. However, this study was conducted on a very small dataset of 705 images. Other methods reported in the literature are found overall less accurate than the proposed system. In our experiment, for ResNet50 the observed accuracy, sensitivity and specificity values are respectively 97.2%, 97.3% 97.1%. For InceptionResNetv2 these values are respectively 97.2%, 96.3%, and 97.5%.

With comparable or better performance, the proposed system produces significantly faster decisions and drastically minimizes the resource requirements. For example, the proposed system requires 12 times less memory in comparison to ResNes50, and produces 2 times faster decisions.

Extensive experiments in this study also reveals some new findings into the literature that include:

1. Low computationally intensive CNNs, specifically, MobileNetV2 and MobileNetV3Small, are capable to detect glaucoma in color fundus photographs with comparable performance with the high computationally intensive CNNs.
2. Glaucoma could be detected more accurately by the CNNs, when only the ONH region rather than the whole image is fed to the models.

In future we would like to evaluate the performance of the system on resource limited hardware.

## Data Availability

I data used in this study is publicly available for research and development from the following sources: LAG dataset: https://paperswithcode.com/dataset/lag, ACRIMA: https://figshare.com/s/c2d31f850af14c5b5232, Drishti-GS: https://cvit.iiit.ac.in/projects/mip/drishti-gs/mip-dataset2/Home.php, HRF: https://www5.cs.fau.de/research/data/fundus-images/, RIM-ONE r2: http://medimrg.webs.ull.es/research/retinal-imaging/rim-one/, sjchoi86 HRF: https://github.com/yiweichen04/retina_dataset, DRIONS-DB: https://www.researchgate.net/publication/326460478_Glaucoma_dataset_-_DRIONS-DB.

## Code availability

The code of this study is available from the corresponding author based on reasonable request and subject to the rule/regulatory approval of the institute.

## References

[1] Quigley HA, Broman AT (2006). The number of people with glaucoma worldwide in 2010 and 2020. Br. J. Ophthalmol..

[2] Costagliola C, Dell’Omo R, Romano MR, Rinaldi M, Zeppa L, Parmeggiani F (2009). Pharmacotherapy of intraocular pressure: Part I. Parasympathomimetic, sympathomimetic and sympatholytics. Expert Opin. Pharmacother..

[3] Bourne RR, Jahanbakhsh K, Boden C, Zangwill LM, Hoffmann EM, Medeiros FA, Weinreb RN, Sample PA (2007). Reproducibility of Visual Field Endpoint Criteria for SAP, FT and SITA strategies: Diagnostic Innovations in Glaucoma Study (DIGS). Am. J. Ophthalmol..

[4] Huang AS, Penteado RC, Saha SK, Do JL, Ngai P, Hu Z, Weinreb RN (2018). Fluorescein aqueous angiography in live normal human eyes. J. Glaucoma.

[5] VISION 2020 Global Initiative for the Elimination of Avoidable Blindness: Action plan 2006–2011 (World Health Organization, 2007).

[6] Tham Y-C (2014). Global prevalence of glaucoma and projections of glaucoma burden through 2040. Ophthalmology.

[7] Tham YC, Li X, Wong TY, Quigley HA, Aung T, Cheng CY (2014). Global prevalence of glaucoma and projections of glaucoma burden through 2040: A systematic review and meta-analysis. Ophthalmology.

[8] Kingman S (2004). Glaucoma is second leading cause of blindness globally. Bull. World Health Organ..

[9] Kolář R, Jan J (2008). Detection of glaucomatous eye via color fundus images using fractal dimensions. Radioengineering.

[10] Michelson G, Warntges S, Hornegger J, Lausen B (2008). The papilla as screening parameter for early diagnosis of glaucoma. Deutsches Arzteblatt Int..

[11] Sivaswamy J, Chakravarty A, Datt Joshi G, Abbas Syed T (2015). A comprehensive retinal image dataset for the assessment of glaucoma from the optic nerve head analysis. JSM Biomed. Imaging Data Pap..

[12] Shoukat, A. & Akbar, S. Artificial intelligence techniques for glaucoma detection through retinal images. In *Artificial Intelligence and Internet of Things* (2021).

[13] Reguant R, Brunak S, Saha S (2021). Understanding inherent image features in CNN-based assessment of diabetic retinopathy. Sci. Rep..

[14] Diaz-Pinto A, Morales S, Naranjo V, Köhler T, Mossi JM, Navea A (2019). CNNs for automatic glaucoma assessment using fundus images: An extensive validation. Biomed. Eng. Online.

[15] Vaghjiani, D., Saha, S., Connan, Y., Frost, S. & Kanagasingam, Y. Visualizing and understanding inherent image features in CNN-based glaucoma detection. In *2020 Digital Image Computing: Techniques and Applications *(*DICTA*) 1–3 (IEEE, 2020).

[16] Almazroa A, Sun W, Alodhayb S, Raahemifar K, Lakshminarayanan V (2017). Optic disc segmentation for glaucoma screening system using fundus images. Clin. Ophthalmol..

[17] Sundaram R, Ks R, Jayaraman P (2019). Extraction of blood vessels in fundus images of retina through hybrid segmentation approach. Mathematics..

[18] Septiarini A, Khairina DM, Kridalaksana AH, Hamdani H (2018). Automatic glaucoma detection method applying a statistical approach to fundus images. Healthc. Inform. Res..

[19] Maheshwari S, Pachori RB, Kanhangad V, Bhandary SV, Acharya UR (2017). Iterative variational mode decomposition based automated detection of glaucoma using fundus images. Comput. Biol. Med..

[20] Agrawal DK, Kirar BS, Pachori RB (2019). Automated glaucoma detection using quasi-bivariate variational mode decomposition from fundus images. IET Image Process..

[21] Kirar BS, Agrawal DK (2019). Computer aided diagnosis of glaucoma using discrete and empirical wavelet transform from fundus images. IET Image Process..

[22] Gour N, Khanna P (2019). Automated glaucoma detection using GIST and pyramid histogram of oriented gradients (PHOG) descriptors. Pattern Recognit. Lett..

[23] Mookiah MR, Acharya UR, Lim CM, Petznick A, Suri JS (2012). Data mining technique for automated diagnosis of glaucoma using higher order spectra and wavelet energy features. Knowl. Based Syst..

[24] Sundaram R, Ravichandran KS (2019). An automated eye disease prediction system using bag of visual words and support vector machine. J. Intell. Fuzzy Syst..

[25] Li Z, He Y, Keel S, Meng W, Chang RT, He M (2018). Efficacy of a deep learning system for detecting glaucomatous optic neuropathy based on color fundus photographs. Ophthalmology.

[26] Christopher M, Belghith A, Bowd C, Proudfoot JA, Goldbaum MH, Weinreb RN, Girkin CA, Liebmann JM, Zangwill LM (2018). Performance of deep learning architectures and transfer learning for detecting glaucomatous optic neuropathy in fundus photographs. Sci. Rep..

[27] Raghavendra U, Fujita H, Bhandary SV, Gudigar A, Tan JH, Acharya UR (2018). Deep convolution neural network for accurate diagnosis of glaucoma using digital fundus images. Inf. Sci..

[28] Al-Bander B, Williams BM, Al-Nuaimy W, Al-Taee MA, Pratt H, Zheng Y (2018). Dense fully convolutional segmentation of the optic disc and cup in colour fundus for glaucoma diagnosis. Symmetry.

[29] Pal A, Moorthy MR, Shahina A (2018). G-Eyenet: A convolutional autoencoding classifier framework for the detection of glaucoma from retinal fundus images. Proc. Int. Conf. Image Process. ICIP.

[30] Liu H, Li L, Wormstone IM, Qiao C, Zhang C, Liu P, Li S, Wang H, Mou D, Pang R, Yang D (2019). Development and validation of a deep learning system to detect glaucomatous optic neuropathy using fundus photographs. JAMA Ophthalmol..

[31] Gómez-Valverde JJ, Antón A, Fatti G, Liefers B, Herranz A, Santos A, Sánchez CI, Ledesma-Carbayo MJ (2019). Automatic glaucoma classification using color fundus images based on convolutional neural networks and transfer learning. Biomed. Opt. Express.

[32] Phan S, Satoh S, Yoda Y, Kashiwagi K, Oshika T, Kashiwagi K, Miyake M, Sakamoto T, Yoshitomi T, Inatani M, Yamamoto T, Sugiyama K, Nakamura M, Tsujikawa A, Sotozono C, Sonoda KH, Terasaki H, Ogura Y, Hiyoyuki I, The Japan Ocular Imaging Registry Research Group (2019). Evaluation of deep convolutional neural networks for glaucoma detection. Jpn. J. Ophthalmol..

[33] Elangovan P, Nath MK (2020). Glaucoma assessment from color fundus images using convolutional neural network. Int. J. Imaging Syst. Technol..

[34] Sreng S, Maneerat N, Hamamoto K, Win KY (2020). Deep learning for optic disc segmentation and glaucoma diagnosis on retinal images. Appl. Sci..

[35] Srinivasa, J., Deekshitha, S., Sushil, U., Dhiya, N. & Kumar, N. S. A high performance glaucoma screening technique using CNN architecture. In *Proceedings of the Fist International Conference on Advanced Scientific Innovation in Science, Engineering and Technology, ICASISET 2020*. 10.4108/eai.16-5-2020.2304033 (2021).

[36] Gheisari S, Shariflou S, Phu J, Kennedy PJ, Agar A, Kalloniatis M, Golzan SM (2021). A combined convolutional and recurrent neural network for enhanced glaucoma detection. Sci. Rep..

[37] Maheshwari, S. & Kumar, T. S. A comparison of local descriptor-based data augmentation techniques for glaucoma detection using retinal fundus images. In *2022 E-Health and Bioengineering Conference* (*EHB*) 01–04 (IEEE, 2022).

[38] Chaudhary PK, Pachori RB (2021). Automatic diagnosis of glaucoma using two-dimensional Fourier–Bessel series expansion based empirical wavelet transform. Biomed. Signal Process. Control.

[39] de Sales Carvalho NR, Rodrigues MD, de Carvalho Filho AO, Mathew MJ (2021). Automatic method for glaucoma diagnosis using a three-dimensional convoluted neural network. Neurocomputing.

[40] Lin M, Hou B, Liu L, Gordon M, Kass M, Wang F, Van Tassel SH, Peng Y (2022). Automated diagnosing primary open-angle glaucoma from fundus image by simulating human’s grading with deep learning. Sci. Rep..

[41] Fan R, Alipour K, Bowd C, Christopher M, Brye N, Proudfoot JA, Goldbaum MH, Belghith A, Girkin CA, Fazio MA, Liebmann JM (2023). Detecting glaucoma from fundus photographs using deep learning without convolutions: Transformer for improved generalization. Ophthalmol. Sci..

[42] Shoukat A, Akbar S, Hassan SA, Iqbal S, Mehmood A, Ilyas QM (2023). Automatic diagnosis of glaucoma from retinal images using deep learning approach. Diagnostics.

[43] Velpula VK, Sharma LD (2023). Multi-stage glaucoma classification using pre-trained convolutional neural networks and voting-based classifier fusion. Front. Physiol..

[44] Li L, Xu M, Liu H, Li Y, Wang X, Jiang L, Wang Z, Fan X, Wang N (2019). A large-scale database and a CNN model for attention-based glaucoma detection. IEEE Trans. Med. Imaging.

[45] Sivaswamy, J., Krishnadas, S. R., Joshi, G. D., Jain, M. & Tabish, A. U. Drishti-gs: Retinal image dataset for optic nerve head (ONH) segmentation. In *2014 IEEE 11th International Symposium on Biomedical Imaging* (*ISBI*) 53–56 (IEEE, 2014).

[46] Budai A, Bock R, Maier A, Hornegger J, Michelson G (2013). Robust vessel segmentation in fundus images. Int. J. Biomed. Imaging.

[47] Fumero, F., Alayón, S., Sanchez, J. L., Sigut, J. & Gonzalez-Hernandez, M. RIM-ONE: An open retinal image database for optic nerve evaluation. In *2011 24th International Symposium on Computer-Based Medical Systems* (CBMS) 1–6 (IEEE).

[48] sjchoi86-HRF Database. https://github.com/sjchoi86/retina_dataset/tree/master/dataset. Accessed 02 July 2017.

[49] Carmona EJ, Rincón M, García-Feijoó J, Martínez-de-la-Casa JM (2008). Identification of the optic nerve head with genetic algorithms. Artif. Intell. Med..

[50] Redmon, J., Divvala, S., Girshick, R. & Farhadi, A. You only look once: Unified, real-time object detection. In *Proceedings of the IEEE Conference on Computer Vision and Pattern Recognition* 779–788 (2016).

[51] Howard, A., Sandler, M., Chu, G., Chen, L. C., Chen, B., Tan, M., Wang, W., Zhu, Y., Pang, R., Vasudevan, V. & Le, Q. V. Searching for mobilenetv3. In *Proceedings of the IEEE/CVF International Conference on Computer Vision* 1314–1324 (2019).

[52] Ren S, He K, Girshick R, Sun J. Faster r-cnn: Towards real-time object detection with region proposal networks. In *Advances in Neural Information Processing Systems* (2015). 10.1109/TPAMI.2016.2577031 27295650

[53] Wong, A., Famuori, M., Shafiee, M. J., Li, F., Chwyl, B. & Chung, J. Yolo nano: a highly compact you only look once convolutional neural network for object detection. In *2019 Fifth Workshop on Energy Efficient Machine Learning and Cognitive Computing-NeurIPS Edition* (*EMC2-NIPS*) 22–25 (IEEE, 2019).

[54] Jocher Glenn. Code Repository. https://github.com/ultralytics/yolov5. Accessed 1 Sep 2023.

[55] Wang, C. Y., Bochkovskiy, A. & Liao, H. Y. YOLOv7: Trainable bag-of-freebies sets new state-of-the-art for real-time object detectors. In *Proceedings of the IEEE/CVF Conference on Computer Vision and Pattern Recognition* 7464–7475 (2023).

[56] Tian Y, Zhao D, Wang T (2022). An improved YOLO Nano model for dorsal hand vein detection system. Med. Biol. Eng. Comput..

[57] Tajbakhsh N, Shin JY, Gurudu SR, Hurst RT, Kendall CB, Gotway MB, Liang J (2016). Convolutional neural networks for medical image analysis: Full training or fine tuning?. IEEE Trans. Med. Imaging.

[58] Thakur A, Goldbaum M, Yousefi S (2020). Predicting glaucoma before onset using deep learning. Ophthalmol. Glaucoma.

[59] Thompson AC, Jammal AA, Medeiros FA (2020). A Review of deep learning for screening, diagnosis, and detection of glaucoma progression. Transl. Vis. Sci. Technol..

